# Beyond MACE: a multidimensional approach to outcomes in clinical trials for older adults with stable ischemic heart disease

**DOI:** 10.3389/fcvm.2023.1276370

**Published:** 2023-11-17

**Authors:** Kriti Kalra, Mohamad B. Moumneh, Michael G. Nanna, Abdulla A. Damluji

**Affiliations:** ^1^ Inova Center of Outcomes Research, Inova Heart and Vascular, Fairfax, VA, United States; ^2^ Section of Cardiovascular Medicine, Yale University School of Medicine, New Haven, CT, United States; ^3^ Department of Medicine, Division of Cardiology, Johns Hopkins University School of Medicine, Baltimore, MD, United States

**Keywords:** quality of life, aging, myocardial ischemia, survey and questionnaire, acute coronary syndrome

## Abstract

The global population of older adults is expanding rapidly resulting in a shift towards managing multiple chronic diseases that coexist and may be exacerbated by cardiovascular illness. Stable ischemic heart disease (SIHD) is a predominant contributor to morbidity and mortality in the older adult population. Although results from clinical trials demonstrate that chronological age is a predictor of poor health outcomes, the current management approach remains suboptimal due to insufficient representation of older adults in randomized trials and the inadequate consideration for the interaction between biological aging, concurrent geriatric syndromes, and patient preferences. A shift towards a more patient-centered approach is necessary for appropriately and effectively managing SIHD in the older adult population. In this review, we aim to demonstrate the distinctive needs of older adults who prioritize holistic health outcomes like functional capacity, cognitive abilities, mental health, and quality of life alongside the prevention of major adverse cardiovascular outcomes reported in cardiovascular clinical trials. An individualized, patient-centered approach that involves shared decision-making regarding outcome prioritization is needed when any treatment strategy is being considered. By prioritizing patients and addressing their unique needs for successful aging, we can provide more effective care to a patient population that exhibits the highest cardiovascular risks.

## 1. Introduction

According to the United Nations (1), the world population of adults over 75 years of age is expected to rise by 40% within the next decade, growing from 165 million in 2020 to 231 million in 2030. The life expectancy at 80 years of age is projected to reach 7.8 years by 2025, 8 years by 2030, and 9.1 years by 2050 (1). With these projections, the number of people aged 75 or older is expected to double by 2050, accounting for over half of the total demographic of the older adult population.

In the United States, a similar demographic shift is evident. The Centers for Disease Control and Prevention (CDC) estimate that the number of individuals >80 years will grow from 9.3 million in 2000 to 19.5 million in 2030 (2). The life expectancy at 85 is predicted to increase from 7.1 years in 2017 to 8.4 years in 2060, according to the US Census (3). These trends call for urgent action in appropriately managing therapeutic strategies and interventions for favorable outcomes among older adults (4). Through recent decades, there has been a clear shift in the major disease spectrum (2), with chronic cardiovascular diseases now forming the majority of the comorbidity burden.

Currently, stable ischemic heart disease (SIHD) ranks among the top causes of morbidity and mortality in older patient populations (5–7). There is a projected doubling in the incidence of SIHD in the population aged >75 years in both men and women (5–8). The Global Burden of Diseases, Injuries, and Risk Factors study that assessed diseases with the largest impact on disability-adjusted life years, showed that in patients aged >75 years, SIHD remains the highest contributor for the past three decades (9). According to the recent American Heart Association (AHA) statement on the management of acute coronary syndrome (ACS) in the older adult population, the highest proportion of individuals who are hospitalized for ACS are patients aged 75 and above (10). These patients also tend to be sicker and require more frequent escalation of care at presentation (11). Owing to cardiovascular changes with aging, pre-existing geriatric syndromes, multimorbidity, and the scarcity of evidence on management of SIHD in the older population with multiple chronic conditions, their clinical outcomes in practice remain suboptimal (10).

### 1.1 Aging and SIHD

The initial definition of successful aging by Rowe and Kahn (12) emphasized the concept of disease avoidance and maintaining a disease-free state, thereby excluding most older patients living with multiple chronic conditions. However, the perceptions of successful aging are evolving. Empirical studies demonstrate that older adults frequently equate successful aging with multi-dimensional behavioral and psychosocial factors (13, 14). In a study, Bowling et al. (15) compared a biomedical and a psychosocial model of healthy aging to patient's own perspectives and observed that there was a divergence in what constituted important parameters of healthy aging. The literature consistently demonstrates a misalignment between the physiological or functional model of aging and the perspectives of older patients (16–18), highlighting the importance of incorporating subjective criteria of assessment.

Patient-reported outcomes (PROs) are defined as measures that directly capture patients' perspectives on their health, functional status, symptoms, and QoL (19). PROs encompass multiple domains pertinent to the health of older patients, including but not limited to functional ability and physical health, social and environmental support, religiosity (20), less depressive affective functioning (18), and intact cognition. There is a compelling call to prioritize PRO in cardiovascular care for older patients (21). The American Geriatrics Society (AGS) advocates for clinicians to engage in discussions about health care with patients and caregivers. Decisions should be aligned with patients' health priorities and their health trajectories instead of disease-specific care (22). The Geriatric 5Ms (mind, mobility, medications, multicomplexity, and “matters most to me”) (23) is a communication tool that can be used in the majority of healthcare decisions in older patients.

The number of older patients living with SIHD is rising, but evidence-based therapies in older adult populations remain limited (24). Randomized controlled trials (RCTs) evaluating management strategies have failed to adequately incorporate geriatric syndromes and age-related physical and cognitive confounders in the precepts of care (25, 26). Moreover, these trials have inconsistently defined major adverse cardiovascular events (MACE) and have largely ignored patient preferences while implementing therapeutic management strategies (25, 26). Consequently, relying solely on clinical practice guidelines based on published literature may prove insufficient and may introduce adverse outcomes in the highest-risk populations of older adults (27). To address this, there is a growing need to broaden our focus from preventing MACE outcomes to capturing the heterogeneous aging experiences, redefining health priorities, and evaluating the progression of geriatric syndromes with cardiovascular outcomes (28, 29).

The central objective of this review is to critically evaluate the relationship between PROs and the treatment approach for SIHD in older patients. We will examine the multidimensional aspects of PROs—encompassing functional ability, physical health, social support, mental health, and cognitive status—and their implications on SIHD management. The care objectives of treating older individuals are distinct, and a patient's perspective on these objectives may outweigh the potential benefits of life-extending, evidence-based treatments. Prior to implementing a treatment plan for this patient population, it is essential to evaluate existing body of evidence and conduct a risk-benefit analysis. We also aim to establish a tailored approach to SIHD management by incorporating a patient-centric model that aligns with older patients' specific health priorities, preferences, and unique aging experiences.

*Key Takeaway*

1. *There is a disconnect between traditional biomedical models of aging and older adults' actual experiences and perspectives*.
2. *Current management strategies for SIHD in older patients are insufficient as they do not account for geriatric syndromes, age-related risks, and patient preferences, necessitating a shift towards an individualized, patient-centered approach that reflects the diverse aging experience of these patients*.

## 2. Definition of older adults

In the foundational framework of active aging, the World Health Organization (WHO) proposed that older adults be defined as individuals over the age of 60 years (30). Despite acknowledging the potential ambiguity of this definition, it became an anchor for the initial age-related frameworks (31). In contemporary times, for both national and international population demographics, the definition of older adults has been extended to include those above 65 years of age consistent with Medicare eligibility (32, 33). Yet, in recent years, as life expectancy has increased, simultaneously with improvements in QoL, there has been an emerging consensus on the need to reevaluate and redefine the definition of older adults. In the Journal of Aging and Physical Activity, there was advocacy for adopting a more nuanced and stratified classification of the older patients (34). This proposed classification by *Spirduso* et al. subdivides the older population into the “young old” (ages 65–74), the “old” (ages 75–84), the “old-old” (ages 85–99), and the “oldest old” (ages 100+). This approach helps to capture the heterogeneity within the older adult population, acknowledging that a 65-year-old and an 80-year-old are likely to have markedly different health and functional profiles and hence have different biological ages.

The concept of biological aging can significantly diverge from chronological aging, which follows a fixed and linear pattern. The aging process is inherently heterogeneous, impacting cellular structures, molecular pathways, and entire organ systems in diverse ways. This complexity arises from various factors, including immune aging, accumulative metabolic damage at the cellular level (35), and inflammageing (increasingly recognized as both a symptom and a cause of age-associated illnesses) (36–38). The emerging understanding of cellular senescence further contributes to this multifaceted process (39). Lipsitz's review encapsulates this dynamic interplay by elucidating that aging is not only associated with increased complexity within anatomic structures and physiological functions, but also with heightened variability in physiological responses (40). This increased variability, coupled with a concurrent decline in adaptive capacity, is a distinctive hallmark of the aging process (40).

The molecular intricacies of aging are further complicated by the impact of diverse factors, including genetics, lifestyle choices, disease burden, and the presence or absence of geriatric syndromes. Geriatric syndromes, such as frailty, multimorbidity, and functional disability, can significantly accelerate the biological and, subsequently, cardiovascular aging processes (41, 42). They also shape the individual's physical resilience or susceptibility to various stressors and diseases. Frailty, a clinical syndrome reflecting a decline in physiological and functional reserve, increases with age and is a precursor of disability (43, 44). Frailty not only contributes to accelerated physiological aging but is independently associated with poor health outcomes regardless of the presence or absence of a specific disease state (45, 46). Another geriatric syndrome, multimorbidity, is highly prevalent in the older population (47–49). It is characterized by the coexistence of diseases that are functionally and physiologically independent but may synergistically contribute to physical dysfunction and functional decline (50, 51). This concept differs from comorbidity, which refers to a condition where one disease state is the chronological successor of multiple interacting conditions (52). As *Calderón-Larrañaga et al*. discuss comprehensively (53), multimorbidity involves a deleterious cycle wherein co-existing diseases interact, thereby undermining compensatory mechanisms and leading to physical and cognitive decline. Conversely, physical and cognitive impairments exacerbate the severity and burden of multimorbidity, thus establishing a bidirectional dysfunction (46). Much like frailty, the presence of multimorbidity is associated with poor functional ability, adverse health outcomes, and increased mortality (48, 49). Taken together, understanding and accounting for these differences is important in delivering effective care to older patients (54). Owing to the significant differences that exist in biological aging with every decade, it is important to revise the definition of “older adult” to encompass a more nuanced approach to aging. In this review, we refer to older individuals as those above the age of 75 and limit our discussion to this cohort.

*Key Takeaway*

1. *The conventional definition of older adults as individuals over 60 or 65 is overly simplistic because it fails to take into account the complexities of biological aging*.
2. *Geriatric syndromes can influence the biological and cardiovascular aging process, impact resilience, and alter an individual's functional status. Hence, a tailored healthcare approach that accounts for these complexities is important*.

## 3. Cardiovascular physiology and aging

A constellation of molecular, biological, and clinical changes constitute the hallmarks of aging that increase the susceptibility of older patients to the spectrum of cardiovascular diseases (55, 56). At the cellular level, multiple interdependent mechanisms and processes are observed that facilitate cardiovascular aging (55, 56). This includes the superoxide-driven upsurge in oxidative stress, chronic low-grade inflammation (57), and the increased expression of pro-inflammatory cytokines (58). Endothelial damage further leads to a dysregulated response to vascular injuries and stressors, impaired vasodilatory mechanisms, and increased intimal thickness (58–61). This detrimental sequence of events leads to a distinctive phenomenon of vascular aging (61–63). Simultaneously, aging also impairs the compensatory mechanism of the cardiovascular system to both internal and external stressors (59). For instance, older patients have impaired myocardial reperfusion post-acute myocardial infarction (AMI), which prolongs the recovery process (60). These cumulative maladaptive structural and functional transformations not only amplify the incidence of SIHD and AMI in older populations but also contribute to poor health outcomes (59). This has been corroborated by observations from several clinical studies. Early research from the GUSTO-I (64) trial highlighted age as a key determinant of outcomes in STEMI patients, a finding supported by the PURSUIT Trial of NSTEMI patients (65). Subsequently, both the Thrombolysis in Myocardial Infarction (TIMI) score (66) and the GRACE score (67) incorporated older age as an important factor that predicts death and cardiac ischemic events. More recently, *Luca* et al. recognized age as the strongest predictor of poor outcomes, even when adjusted for other risk factors (68). Both Rosengren et al. *(*69), and APEX-AMI (70) showed that in patients with ACS and STEMI, respectively, there was an increased likelihood of heart failure, cardiogenic shock, atrial fibrillation, and recurrent ischemia in older patients. In-hospital death rates also markedly increased in proportion to higher age for patients over 75 years. This understanding is important because not only the mechanism of SIHD development is significantly different in older patients, but also the trajectory it follows.

The second confounder of outcomes is the presence of geriatric syndromes, such as frailty, cognitive impairment, and multimorbidity, which amplifies the risk of systemic diseases, including SIHD, in older patients. Geriatric syndromes are associated with poor health outcomes across the spectrum of SIHD severity. The prevalence of frailty in older patients presenting with coronary disease is estimated to be as high as 19% (71). Frailty has been linked to sub-optimal/detrimental cardiovascular and all-cause morbidity and mortality outcomes in older patients with SIHD (54, 71–78). Adding to this burden is the presence of comorbidities which exacerbates the risk of poor health outcomes. Mortality risk in older patients has been shown to increase in a proportional pattern with the increase in comorbidity burden (79–81), and the benefits of revascularization, while generally improving survival after ACS event, show progressive less benefit with increasing comorbidity and frailty burden (79, 82).

Cognitive impairment, which is shown to increase in prevalence with increasing age (83), further complicates the prognosis in older patients with SIHD (46). Even mild cognitive impairment has been associated with poor cardiovascular outcomes (84). A higher 30-day mortality rate and an increased risk of admission at one year were reported among those with dementia or advanced cognitive decline (85). A recent meta-analysis reinforced this concept by demonstrating a higher short-term (30-day) and long-term mortality in older patients with cognitive impairment compared to their cognitively intact peers (86). Hence, an improved understanding of the influence of age-associated factors on MACE outcomes can help improve the clinical management of the older patient populations.

*Key Takeaways*

1. *Chronological age influences SIHD outcome. There is a higher risk of cardiac ischemic events, heart failure, atrial fibrillation, and cardiogenic shock in patients aged 75 years and older*.
2. *The spectrum of changes associated with biological aging and geriatric syndromes, such as frailty, cognitive impairment, and multimorbidity, contribute to poor health outcomes in older patients with SIHD and should be taken into account when planning for any revascularization strategy*.

## 4. Older adults in SIHD trials

The establishment of clinical practice guidelines and their general applicability necessitates evidence based on a well-represented study population. The lack of age-appropriate safety data, adverse event profiles, and insights into real-world effectiveness can, and does, lead to poor clinical outcomes (87). Up until the last decade, the majority of clinical practice guidelines on the prevention, diagnosis, and management of SIHD in the older adults have been extrapolation from clinical trials from the much younger patient cohorts. In fact, a recent systematic review of all clinical trials focusing on ACS management revealed that a meager 12.9% of over 1 million patients enrolled were aged 75 and above (88). Despite efforts to improve enrollment of representative older populations by removing chronologic age cutoffs, progress has remained modest (89).

Several factors contribute to the poor representation of older adults in clinical trials, which can be broadly classified into three categories: restrictive study design, recruitment difficulties, and retention challenges (90). Geriatric syndromes such as frailty, multimorbidity, and cognitive impairment pose significant challenges at each of these stages. The presence of geriatric syndromes is frequently encountered as a study exclusion criteria itself. Multimorbidity poses a challenge for both the internal and external validity of clinical trials (91). It can affect appropriate treatment selection as well as lead to confounding of treatment outcomes (91). Furthermore, polypharmacy is a common occurrence in older adult individuals due to the prevalence of multimorbidity. Polypharmacy complicates the investigation of the efficacy and safety of new drugs due to the higher potential for drug-drug interactions (92), which can lead to ineffectiveness and increased adverse events. Beyond the biological model, geriatric syndromes also impact recruitment and compound retention problems due to various logistical hurdles. These issues span a wide spectrum: transportation and mobility difficulties arising from functional dependence, economic constraints, and limited understanding or access to digital technology, all of which could curtail participation or heighten dropout rates (90). All these factors lead to under-representation, not only by direct causal effects but also by selection biases, as clinicians and researchers hesitate to recruit these complex patients in clinical trials (93).

*Key Takeaways*

1. *Most clinical guidelines on prevention, diagnosis, and management of SIHD have been based on clinical trials primarily involving younger patient cohorts, and hence there is inadequate representation of older adults*.
2. *Selection bias, restrictive study design, recruitment difficulties, retention challenges, and logistical issues have all contributed to the underrepresentation of older adults in cardiovascular clinical trials*
3. *Geriatric syndromes such as multimorbidity, polypharmacy, frailty, and cognitive impairment further complicate clinical trials due to potential interactions*.

## 5. Current outcomes measures in clinical trials

### 5.1. Traditional MACE vs. patient-centered outcomes

Following on the initiatives from the National Institutes of Health, including the Inclusion Across the Lifespan policy (94), there has been a deliberate shift towards inclusion of older patients in cardiovascular trials. However, many of the more recent pivotal trials focused on traditional MACE outcomes such as mortality, rehospitalization, repeat revascularization, stroke, and peripheral vascular disease (68). These outcomes are seldom standardized or take into account the complexity of age-associated conditions that coexist in the older population. Table 1 shows the major landmark trials and registries that are currently inclusive of the older adult population.

**Table 1:** Major randomized clinical trials and prospective cohort studies that enrolled older adult populations with coronary artery disease.

Study	Year	Total Participants	Age	Disease	Treatment strategy under investigation	Primary outcome	Secondary outcome	Extra adverse events studied	Special comments	Geriatric syndromes evaluated (Y/N); Additional comment
GUSTO-I Trial (64)	1996	5,037	78.7 ± 2.6	STEMI	Thrombolytic therapy	All cause 30-day mortality	Stroke, death or stroke, and death or nonfatal, disabling stroke.	Major bleeding, cardiogenic shock, reinfarction, Arrythmia, Heart failure	–	N
SENIOR-PAMI (95)	2005	483	Mean 78 (range 70–101)	STEMI	PCI vs thrombolytic therapy	Death or disabling stroke at 30 days	Death, disabling stroke, or reinfarction at 30 days	–	–	N
Outcomes of Coronary Artery Bypass Graft Surgery (CABG) in Octogenarians (96)	2007	70–79 Years (852, 28.6%); 80 Years (282, 9.4%)	74 ± 3; 83 ± 3	Multivessel CAD	CABG outcomes in different age groups	Hospital mortality, major postoperative complications (perioperative myocardial infarction, respiratory failure, renal failure, deep sternal wound infection, bleeding requiring reoperation, unplanned reoperation, stroke, and gastrointestinal complications), length of hospital stay, and late survival	Individual elements of primary composite outcomes	–	Noted that burden of comorbidity in elderly patient group may negate any benefit on long-term survival gained by improvements in early outcome.	N
PLATO trial (97)	2009	18,624. Only 15.5% patients were =/> 75	62	ACS	Ticagrelor vs Clopidogrel	Time to the first occurrence of composite of death from vascular causes, myocardial infarction, or stroke	Composite of death from any cause	Minor bleeding, dyspnea, bradyarrhythmia, any other clinical adverse event, and results of laboratory safety tests	–	N
TRIANA (98) elderly	2011	266	81	STEMI	Primary PCI vs Fibrinolysis	Composite of all-cause mortality, re-infarction, or disabling stroke at 30 days	Death, re-infarction, or disabling stroke at 1 year	–	–	N
Italian ELDERLY ACS study (99)	2012	313	82	NSTEMI	Early aggressive vs conservative strategy	Composite of all-cause mortality, nonfatal myocardial infarction (MI), disabling stroke, and repeat hospital stay for cardiovascular causes or severe bleeding within 12 months	–	–	–	N
XIMA (100)	2014	800	83.6 ± 3.2 (80–101)	Stable angina, Unstable angina, NSTEMI	DES vs BMS	1-year composite of death, myocardial infarction, cerebrovascular accident, target vessel revascularization, or major hemorrhage.	–	–	–	N
LEADERS FREE (101)	2015	2,432	75.7 ± 9.4	Clinical indication for PCI	Efficacy of polymer-free drug-coated coronary stents in patients at high bleeding risk	Composite of cardiac death, myocardial infarction, or stent thrombosis	Bleeding, target-vessel revascularization, and indexes of technical procedural success.	–	64% patients enrolled had higher risk of bleeding by virtue of age	N
MOSCA (102)	2016	106	83 ± 6	NSTEMI	Invasive vs conservative strategy	Composite of all-cause mortality, recurrent myocardial infarction (new episode of chest pain with troponin elevation after admission, during either the index hospital stays or a new readmission) and readmission for cardiac cause (post discharge revascularization or acute heart failure)	All-cause mortality, the composite of mortality or ischemic events (reinfarction or post-discharge revascularization), and bleeding episodes	–	Noted that although benefits were favorable in short-term, long-term outcomes were insignificant and hence may not be beneficial in elderly population.	Comorbidity burden
After Eighty Study (103)	2016	457	84.7 (80–94)	NSTEMI, Unstable angina	Invasive vs conservative strategy	Composite of myocardial infarction, need for urgent revascularization, stroke, and death—the first occurring event.	Death from any cause	Minor and major bleeding	A change of positive effect of invasive treatment occurs in both magnitude and presumably in direction with increasing age	Depression was considered as risk factor. Noted higher co-morbidity burden in patients in comparison to previous RCTs done on younger population
SENIOR (104)	2018	1,200	81·4 (SD 4·3)	Stable angina, silent ischemia, or an acute coronary syndrome	Drug-eluting vs bare-metal stents with short duration of dual ant- platelet therapy	Composite of all-cause mortality, myocardial infarction, ischemia-driven target lesion revascularization, or stroke	Bleeding complications; definite or probable stent thrombosis; all revascularizations; all components of the primary endpoint; and cardiovascular death, at 30 days, 180 days, 365 days, and 2 years	Bleeding risk with duration of DAPT	Noted that indices of fraility were not taken into account	N
Elderly ACS 2 trial (105)	2018	1,443	80 (77–84)	ACS	Antiplatelet therapy	Composite of all-cause mortality, MI, disabling stroke, and rehospitalization for cardiovascular causes or bleeding within 1 year	Global occurrence of cardiovascular death, MI, and stroke; all-cause mortality, cardiovascular mortality at 1 year, and MI at 1 year; type 2 or 3 bleeding within 12 months; any stroke within 12 months; and total number of days spent in hospital within 12 months after index admission.	Average daily bleeding rates and Average ischemic daily rates over 1 year (Time course of ischemic events)	-	N
AFIRE (106)	2019	2,215	74.3 ± 8.3	ACS + Atrial fibrillation	Anticoagulation monotherapy vs combination therapy	Composite of stroke, systemic embolism, myocardial infarction, unstable angina requiring revascularization, or death from any cause.	Individual components of the primary end point; net adverse clinical events; bleeding events	–	–	N
IMPROVE-IT secondary analysis (107)	2019	2,798	85	ACS	Lipid lowering strategy	Composite of CVD death, major adverse cardiac event (nonfatal MI, unstable angina leading to hospitalization, coronary revascularization after day 30), or nonfatal stroke.	Composite endpoint of death due to all causes, major coronary events, and non-fatal stroke, revascularization	Post hoc safety events included cataracts and adverse neurocognitive events.	–	N
CRUSADE registry (108)	2019	6,893	75 (70–81)	ACS	Beta-blocker use > 3 years	Cardiovascular composite of all-cause death, hospitalization for recurrent MI, hospitalization for ischemic stroke at 3 years	Hospitalization for heart failure over the subsequent 5 years	–	–	N
POPular AGE (109)	2020	1,011	73–81	STEMI	Optimal platelet inhibition	PLATO major or minor bleeding, net clinical benefit of all cause death, MI, stroke and PLATO major or minor bleeding	Individual components from net clinical benefit outcome, and cardiovascular death, definite stent thrombosis, urgent revascularization, unstable angina, and transient ischemic attack.	–	–	N
SWEDEHEART registry (110)	2020	14,005	85.4 (±4.1)	ACS	Ticagrelor vs Clopidogrel	Ischemic outcome (death, MI, or stroke), and bleeding	–	–	–	N
RINCAL (111)	2021	250	85.2 (80 to 95)	NSTEMI	Invasive vs conservative strategy	All-cause mortality and non-fatal myocardial reinfarction at one year post randomization	Time to death or non-fatal reinfarction, unplanned revascularization, permanent stroke, major bleeding during hospital admission and at one year, deterioration in renal function during hospital admission, angina burden at three months and one year and stent thrombosis at one year.	–	Exemplifies the difficulty of recruiting elderly to strategy-based investigations, especially when the baseline risk of the patient cohort is inherently high	N
ICON-1 (112)	2022	267	81.2 ± 4.1	NSTEMI	Outcomes after invasive strategy in frail vs non-frail	Composite of all-cause mortality, MI, stroke, repeat unplanned revascularization, and significant bleeding	Individual elements of primary composite outcomes	–	One of the only clinical trials that take into account one of the most important geriatric syndromes	Y

ACS, Acute Coronary Syndrome; AFIRE, antithrombotic therapy for atrial fibrillation with stable coronary disease study; BMS, bare metal stent; CABG, coronary artery bypass graft surgery; CAD, coronary artery disease; CRUSADE, comparative effectiveness of *β*-blocker use beyond 3 years after myocardial infarction and long-term outcomes among elderly patients registry; CVD, cardiovascular diseases; DAPT, dual anti-platelet therapy; DES, drug eluting stent; Elderly ACS Study, comparison of reduced-dose prasugrel and standard-dose clopidogrel in elderly patients with acute coronary syndromes undergoing early percutaneous revascularization; GUSTO-I Trial, global utilization of streptokinase and TPA for occluded coronary arteries trial; ICON-I, study to improve cardiovascular outcomes in high-risk older patients with acute coronary syndrome; IMPROVE-IT, ezetimibe added to statin therapy after acute coronary syndromes study; Italian ELDERLY ACS Study, early aggressive versus initially conservative treatment in elderly patients with non-ST-segment elevation acute coronary syndrome; LEADERS FREE, prospective randomized comparison of the BioFreedom biolimus A9 drug-coated stent versus the gazelle bare-metal stent in patients at high bleeding risk trial; MOSCA, invasive and conservative strategies in elderly patients with non-STEMI; MOSCA-Frail, invasive and conservative strategies in elderly frail patients with non-STEMI; MI, myocardial infarction; NSTEMI, non- ST-elevation myocardial infarction; PCI, percutaneous coronary intervention; PLATO Study, platelet inhibition and patient outcomes study; PoPular Age, clopidogrel versus ticagrelor or prasugrel in patients aged 70 years or older with non-ST-elevation acute coronary syndrome trial; RINCAL, revascularisation or medical therapy in elderly patients with acute anginal syndromes trial; SD, standard deviation; SENIOR, drug-eluting stents in elderly patients with coronary artery disease study; SENIOR-PAMI, senior primary angioplasty in myocardial infarction study; STEMI, ST-elevation myocardial infarction; SWEDEHEART, Swedish Web-system for enhancement and development of evidence-based care in heart disease evaluated according to recommended therapies registry; TRIANA elderly, TRatamiento del Infarto Agudo de miocardio eN Ancianos study (primary angioplasty vs. fibrinolysis in very old patients with acute myocardial infarction); XIMA, Xience or vision stents for the management of angina in the elderly study.

When we examine these trials, it is strikingly clear that the participation of older patients in clinical trials remains unsatisfactory. This underrepresentation is disproportional to the prevalence of SIHD in this population. The definition of MACE as a primary or co-primary outcome also exhibits considerable variability across the trials, and the follow-up varies significantly. These inconsistencies pose a significant challenge in pooling the data and comparing the results for generalizability. Moreover, geriatric syndromes have rarely been evaluated in landmark trials, except After Eighty Study (103) and ICON-1 (112). These parameters significantly impact the prognosis and management of SIHD for older individuals.

Even with recent strides that have been made to better understand and address the health outcomes pertinent to older patients, an essential facet that remains inadequately addressed is considering patient-centric preferences. These preferences, shaped by their own perception of successful or healthy aging, can deviate from the traditionally disease-oriented view of clinicians, researchers, and/or policymakers. For instance, von Faber et al. (113) presented a model of successful aging that includes optimal physical and social functioning as well as the subjective state of well-being. In their study cohort of individuals aged over 85 years, while only 10% of patients met the traditional health metrics of successful aging, a striking 80% subjectively reported successful aging. This perspective highlights that older patients often view successful aging as an adaptive process that consists of physical and social functioning and may not ascertain all benefits in traditional terms. Strawbridge et al. (114) compared patients' self-rating with Rowe and Kahn's criteria of successful aging (12). Only 18% of patients met the criteria as defined by Rowe, but >50% rated themselves as successful aged. They found that the self-rated model of successful aging demonstrated stronger associations with most well-being measures when compared to the Rowe and Kahn model.

**Figure 1:**
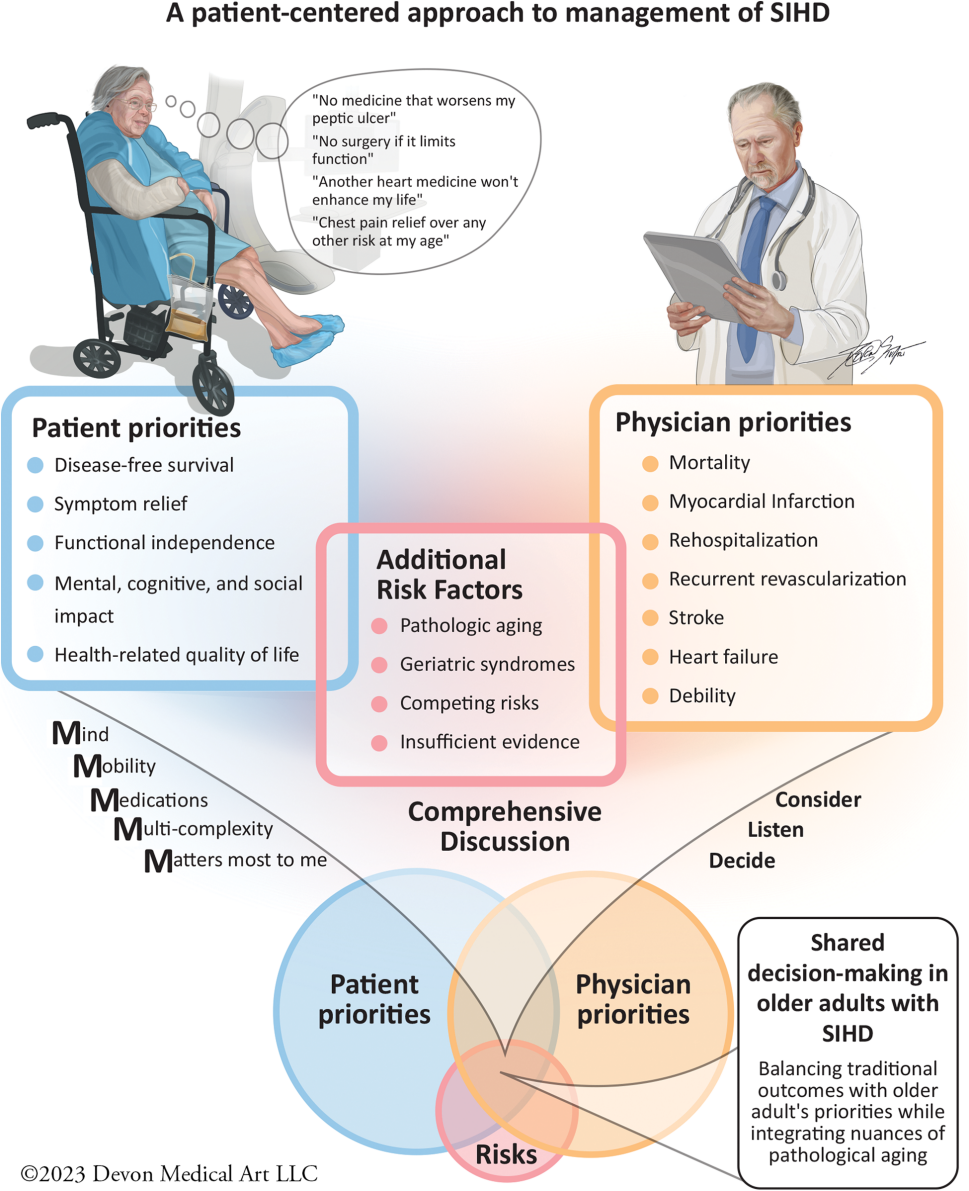
In older adults presenting with SIHD symptoms, it is essential to first identify their expected outcomes and set priorities in the context of potential competing results. Physicians then must take into account the additional risk factors, such as age, concomitant geriatric syndromes, and evaluate the evidence supporting specific therapies. Physicians then present management options and highlight their associated traditional outcomes, such as MACE to the patient. Using the “Geriatric 5 M's” and the “Consider, Listen, and Decide” Approach, can help to promote shared decision-making. This approach ensures we develop a strategy that respects patient-reported outcomes and aligns with evidence-based therapy for SIHD management.

Montgomery and Fahey (115) noted the divergence between patients' and physicians' treatment preferences in the importance of healthcare outcomes. Patients were more likely to select an additional therapeutic intervention if they perceived a significantly elevated disease-related risk, although the focus was on a single disease-related outcome. For older SIHD patients, this decision-making process becomes even more complex due to the multimorbidity (116) and polypharmacy (117). Nanna et al. identified that age influences treatment goals, willingness to consider invasive cardiac procedures, and risk tolerance among hospitalized older patients with SIHD. As patients age, they tend to more frequently prioritize goals, such as maintaining independence and mental capabilities, while being concerned with the loss of physical abilities and mental capacity (118). The AGS has acknowledged the importance of “preference-sensitive” decisions (117), stating that outcomes valued by older patients may deviate from disease-focused clinical practice guidelines and, in fact, may be in conflict with their individual health preferences. Fried et al. *(*119) examined the concept of competing outcomes of significant relevance in the older population. When queried about single-disease treatment preferences, patients would initially strongly align with evidence-based guidelines. However, a notable shift was observed when their global health was considered: they prioritized avoiding significant adverse effects secondary to therapy over disease-focused treatment guidelines. This was especially pronounced when treatment had marginal effects on disease-specific outcomes and/or failed to improve their QoL. This trend was in alignment with a subsequent study wherein more than 90% of the patients would decline even a low-burden evidence-based therapy if it potentially led to functional or cognitive impairment, irrespective of disease-specific benefits (120). Examining cardiovascular outcomes specifically, Tinetti et al. (121) reported that nearly half of their older cohort prioritized mitigating the risk of fall injuries and medication-related symptoms over curtailing the future risk of cardiovascular events in hypertension management. In a similar way, Caughey et al. (122) found that the initial patient preference for taking a disease-specific medication dropped dramatically when potential adverse events or competing health outcomes were considered. The inclusion of these preferences enables clinicians and investigators to evaluate the comprehensive impact of any treatment strategy on the overall health and QoL of older adults from their own perspective. Nanna et al. have recommended a “Consider, Listen, Decide” approach to complex decision-making in older adults with SIHD that incorporates these concepts (24, 123). We discuss the important patient-reported outcomes and their relevance in managing CAD in the older patient population below (Figure 1).

*Key Takeaways*

1. *Traditional MACE outcomes, such as rehospitalization, stroke, and mortality, that are often used in clinical trials are seldom standardized in accordance with pre-existing conditions in older adults*.
2. *Older patients often view successful aging as an adaptive process that includes optimal physical and social functioning rather than meeting traditional health metrics. Older adults may prioritize avoiding significant adverse effects secondary to therapy over disease-focused treatment guidelines, especially when the treatment has marginal effects on improving their QoL*.
3. *Competing health outcomes is an important consideration for older patients with multimorbidity and polypharmacy*.

### 5.2. Patient-reported outcomes

#### 5.2.1 Quality of life

The WHO defines QoL as “an individual's perception of their position in life in the context of the culture and value systems in which they live and in relation to their goals, expectations, standards, and concerns” (124). Bowling et al. defined QoL as a concept that is a collection of interactive objective and subjective dimensions (125). Bowling further refined the definition as a measure that reflects not just macro-societal influences but also delves into the nuances of an individual's personal experiences, circumstances, well-being, values, perceptions, and self-assessment of health (125).

One of the earliest large-scale oL assessments that involved patients' own perspectives was done by posing open-ended questions to patients eliciting a multifaceted range of responses that spanned several domains: independence, social relationships, social roles and activities, health, psychological well-being, perspective about home and neighborhood, and financial circumstances (125). In addition to identifying the core values that defined the meaning of QoL for older patients, this study also demonstrated the wide array of responses elicited by similar questions of integrating individualistic preferences. A public survey conducted by Brown et al. *(*126) identified the most important components of QoL for older patients—family and social relationships, emotional well-being, spirituality, functional independence, social engagement, standard of living, and health maintenance. Multiple studies focusing on older men and women further corroborated these QoL facets (121, 127, 128).

The QoL outcomes tie into what constitutes successful aging for older cardiovascular patients. This also makes it important to factor in the phenomenon of response shift, a concept that elucidates changes in an individual's QoL perception based on alterations in their internal standards, values, or conceptualizations (129). Patients' present QoL preferences might evolve over time in line with their health and life trajectory. Hence, the emphasis on long-term QoL outcomes is critical in concordance with the discussion of short-term benefits. Owing to these insights, the focus has appropriately shifted toward QoL measures in older patients, and numerous global policies and decisions are being implemented to enhance QoL with a focus on long-term care (21, 22, 32). In the context of SIHD management, the inclusion of QoL outcomes remains critical. The symptoms and impacts of the disease—such as chest pain, shortness of breath, and fatigue—can greatly influence both QoL and Health-related quality of life (HRQoL), as these factors often limit social activities, induce emotional distress, and affect overall well-being. Accurately assessing QoL outcomes in the context of cardiovascular treatment therapeutics improves patient selection, helps provide a roadmap for discussions regarding the risks and benefits of treatment, and solidifies a process of shared medical decision-making, particularly for invasive cardiovascular procedures. The instruments used to measure patients' preference for QoL that are validated in older patients are described in Table 2.

**Table 2:** Tools to measure patient reported quality of life outcomes.

Tool	Domains and description	Pros	Cons
WHOQOL OLD (130–132)	It covers six facets: sensory abilities; autonomy; past, present, and future activities; social participation; death and dying; and intimacy.	Specifically designed for older adults. Covers unique aspects of aging, such as autonomy and social participation.	Longer than EQ 5D and SF 36. Needs to be administered along with WHOQOL 100 or WHOQOL BREF.
ASCOT (Adult Social Care Outcomes Toolkit) (133)	Has domains which assess individuals’: control over their daily life, personal care, eating habits, living conditions, safety, social situation, leisure time, self-care and health awareness	Assesses access to social services, and social care-related quality of life.	Does not take into consideration disease-specific measures
ICECAP-O (ICE pop CAPability measure for Older people) (134)	Five conceptual attributes are assessed: attachment, role in society, enjoyment, security, and control	Focuses on individuals’ own perceptions of their capabilities, rather than providing some notion of an objective assessment of capability (135)	May be more responsive to mental health-related changes than physical health due to the domains assessed. (136)

EQ-5D, euro-quality of life 5 dimension instrument; SF-36, short form health survey; WHOQOL 100, World Health Organization quality of life 100 questions; WHOQOL BREF, World Health Organization quality of life brief version; WHOQOL OLD, World Health Organization quality of life for older adults.

*Key Takeaways*

1. *QoL is a multidimensional concept that includes macro societal influences as well as individual experiences, circumstances, well-being, values, and perceptions. This encompasses components such as family, social relationships, emotional wellbeing, spirituality, functional dependence, the standard of living, and health maintenance*.
2. *The concept of QoL aligns with the self-perception of successful aging for older cardiovascular patients and hence is an important outcome to factor in for any management strategy*.
3. *There is a lack of standardization and validation of tools to measure patient-centered QoL in older CAD patients*.

#### 5.2.2. Health-related quality of life

The CDC defines HRQoL at the individual level as perceptions of physical and mental health, such as energy levels, mood, and their correlates (137). For all individuals, especially older people, health impacts not only their functioning but their global QoL, and hence, maintaining good health and minimizing its disease impacts is one of the most important preferences (138). There exists a bidirectional interaction between unrelated symptoms, functionality, and the resultant QoL, which can help in HRQoL assessment (139).

The critical role of HRQoL assessment is its ability to influence disease outcomes positively and guide treatment strategies (140, 141). To address HRQoL assessment, the CDC proposed four core values: patients' perspectives on general and physical health, mental health, and the impact of poor physical or mental health on their usual activities (137, 142). These core values are foundational for the creation of the Patient-Reported Outcomes Measurement Information System (PROMIS) database, which was a landmark step towards incorporating patient perspectives in HRQoL measurement and laid the groundwork for validating all patient-reported outcomes (143).

However, when measuring HRQoL, we must consider the heterogeneity of the tools used for measurement. While tools like SF-36 and EQ-5D-5l offer a broad, encompassing perspective on HRQoL, they may lack the sensitivity to capture disease-specific subtleties. In contrast, the HUI-III, despite its less widespread use, can provide nuanced functional status and coping assessments, which could provide valuable insights in the context of SIHD (144, 145). An early assessment comparing six widely used generic instruments against SIHD-specific HRQoL tools found that generic tools were capable of measuring both SIHD-specific symptoms and their impact on global health along with patients' overall health status (146). Table 3 discusses various generic and SIHD-specific/validated HRQoL tools.

**Table 3:** Tools to measure patient reported health-related quality of life outcomes.

Tool	Description	Pros	Cons	Other forms	Relevance to CAD
SF 36 (Short Form Health Survey) (147)	A 36-item, patient-reported survey of patient health. Measures eight health domains: physical functioning, role limitations due to physical health, bodily pain, general health perceptions, vitality, social functioning, role limitations due to emotional problems, and mental health.	Widely used and validated (145), including in older patients (148). Sensitive to changes in health status. Includes the notion of positive health. Can be used in cost-utility studies (149).	Can be time-consuming for patients. May be less sensitive to specific disease-related issues as the scoring is done in two major groups: physical health and mental health and the scores cannot be combined to get one health index.	Multiple shorter sub-sets have been validated with similar efficacy: SF-12 (150–152), SF-8, and SF-6D. SF-12 has been validated in CAD patients (153).	SF-36 has been validated in multiple studies for CAD patients (154, 155). More floor effect in CVD patients: is more sensitive in milder forms of the disease (156).
EQ -5D- 5l (EuroQol five dimensions questionnaire) (157)	A standardized instrument for measuring generic health status. Represents 5 health domains: Pain, mood, mobility, self-care and daily activities.	Simple and quick to complete. Generates a self-rated assessment of health status on a visual analog scale. Provides a single index value, and utility values with a higher range. Suitable for cost-effectiveness studies, and cost-utility analysis.	Limited sensitivity to small changes in health status. Higher ceiling effects.	EQ-5D-3l is shorter version	Validated for reliability in CAD. Most commonly used preference-based measure in CAD studies (155, 158). A higher ceiling effect in CVD patients is noted (156, 159, 160).
SIP (Sickness Impact Profile) (161)	Is a descriptive analysis with 3 major groups and 12 categories. The major domains assessed are physical dimension, psychosocial dimension and independent categories such as sleep, eating work.	Can be done both by healthcare worker, patients themselves as well as patient proxy	Complex, tedious.	Shorter version: SIP 68	Although generic, has been used in CAD patients, although not extensively (162).
HUI-III (Health Utilities Index—III) (144)	Eight components are assessed: vision, hearing, speech, ambulation, dexterity, emotion, cognition, pain	Can be both self-administered and conducted via interviews. Provides a single index value. Can be used for cost-utility, health-utility measures.	Does not include any geriatric syndrome. More focused on functional status.	Older versions include HUI 1, HUI 2	Validated in CAD patients
QWB (Quality of Well Being Scale)-Self administered (163)	Includes five sections: presence/absence of chronic disease which include acute physical symptoms as well as mental health symptoms and behaviors; mobility; physical activity and social activity	Responsive to change resulting from treatment interventions	Lengthy and time consuming to complete, about 10–15 min.	–	The QWB can be used to measure health-related quality of life in CAD patients, including the impact of physical and social activities (146, 164).
NHP (Nottingham Health Profile)	Comprised of two parts- the first part asks yes/no questions on six scales: mobility, pain, energy, sleep, emotional reactions, and social isolation; second part assesses the effects of each on domains of daily living	Simple and quick.	Has not yet been validated specifically in elderly patients. Does not provide a comprehensive health assessment. Higher floor and ceiling effects (165).	–	May be inconsistent in grading symptoms of angina and the health burden of severe symptoms (145, 166).
COOP/WONCA charts (The Dartmouth COOP Functional Health Assessment Charts/WONCA) (167)	Set of visual charts that assess the following domains: physical fitness, feelings, daily activities, social activities, changes in health, overall health, and pain.	Validated in elderly population (168). Simple to use and low burden on respondents. Visual format can be helpful for those with literacy barriers.	Less comprehensive than other tools. May have limited sensitivity to small changes	–	Have not been extensively tested in CAD patients.

CAD, coronary artery disease; CVD, cardiovascular diseases; EQ Instrument, Euro-quality of life; SF, short form health survey.

**Figure 2:**
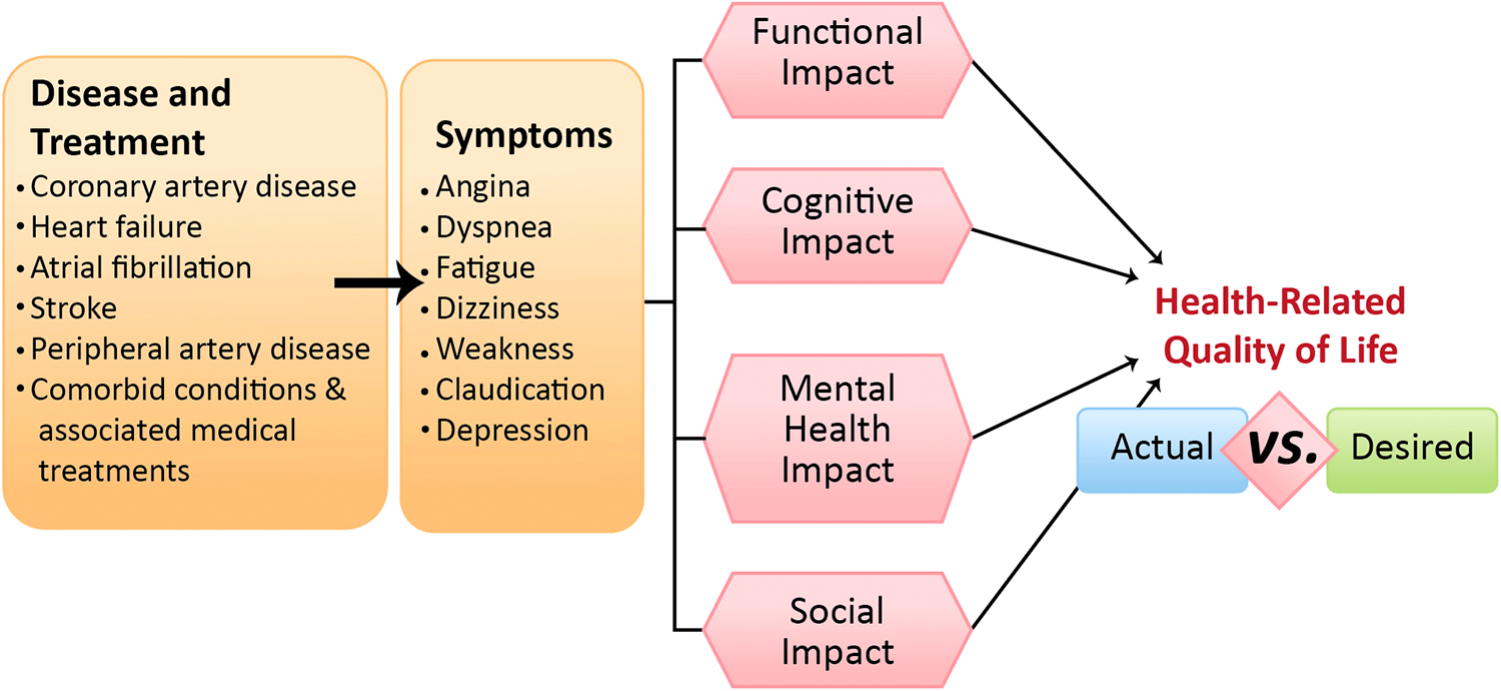
Cardiovascular diseases, associated comorbidities, and multimorbidities can lead to a spectrum of symptoms that may or may not directly reflect direct causation. These initial symptoms are the more obvious cardiovascular symptoms, such as angina, dyspnea, fatigue, etc., but these symptoms, in turn, exert distinct impacts on four key areas: functional, cognitive, mental health, and social domains. Collectively, these influences shape older adults’ health-related quality of life, highlighting a potential disparity between clinical observations and patients’ desires.

These instruments have inherent limitations, often involving trade-offs between comprehensiveness, feasibility, disease specificity, and global health. From a broader perspective, investigators and clinicians must carefully select the most appropriate instrument taking into account their unique strengths and weaknesses. Owing to the diverse study populations with SIHD and the numerous tools of HRQoL utilized in practice, standardization and comparisons of existing instruments are challenging (158, 169). A disease-specific and a generic HRQoL tool together may be required in tailoring treatment plans that prioritize patient preferences and improve overall outcomes in the management of SIHD. As the field evolves, there is a pressing need for robust, replicable, and standardized tools for HRQoL measurement in the context of older patients with SIHD.

*Key Takeaways*

1. *HRQoL determines the impact of health on an individual's perceived well-being, with physical and mental health perceptions as critical components*.
2. *HRQoL assessment can positively impact SIHD outcomes and guide treatment strategies that align with patient preferences*.
3. *There is significant heterogeneity in current HRQoL tools, and standardization is required. Generic tools may not capture SIHD-specific nuances, and hence comprehensive understanding is needed before implementing them in clinical trials and clinical practice*.

#### 5.2.3. Functional status

Age-related functional decline is a widely recognized phenomenon in geriatric medicine (170). As individuals advance in age, their functional capacity, or their ability to conduct daily tasks and activities vital for maintaining independence, tends to diminish. This capacity encompasses both activities of daily living (ADLs), and instrumental activities of daily living (IADLs). In the context of successful aging, preservation of functional ability is often prioritized as a key health outcome by older patients (171–174). Functional independence was identified as an important domain for maintaining the QoL in the Delphi consensus study (175), reflecting the findings of a comprehensive review by van Leeuwen which identified functional independence as the most common domain in patients' perspectives on healthy aging (176). The importance of functional status is further emphasized in settings of multiple diseases and limitations. Any health problem or outcome that hinders an individual's capacity to carry out desired or necessary tasks leads to poor functional outcomes (177). Furthermore, health conditions that limit a patient's daily activities are typically prioritized as severe or urgent among all comorbidities (178). This desire for independence extends beyond basic functional activities and encompasses maintaining adequate mobility, living independently, and continuing work-related activities (173, 179). Functional independence also carries significant protective effects on health. Functional limitations exacerbate feelings of social isolation and can negatively affect HRQoL.

Older patients, particularly those who have suffered from acute health conditions such as MI, often experience a significant decrease in their functional capacity (180). SIHD and related conditions, such as angina pectoris, markedly impair physical activity in older patients. This impairment consequently leads to reduced QoL and further diminishes their functional independence. It's important to note that these negative impacts on functional ability and independence can, in turn, adversely affect PROs, adding another layer of complexity to the management of older patients with SIHD. Therefore, the inclusion of functional status assessment and outcomes in SIHD management strategies for the older adult population is important in aligning care with the patient's health goals.

*Key Takeaways*

1. *Preserving functional ability is one of the most important PROs across older adults' cohort*.
2. *SIHD can impose restriction in functional abilities, which in turn can negatively affect social and mental domains and significantly affect the HRQoL*.
3. *Revascularization strategy that improves functional ability and outcomes that correlate with better functional health, may be prioritized in older adults living with debilitating SIHD*.

#### 5.2.4. Symptoms

In the older population, the burden of symptoms is complex and multifaceted, given the prevalence of multiple chronic conditions. Findings from the National Health and Aging Trends study found that at least 20% of community-dwelling older adults experienced two symptoms concurrently, including pain, fatigue, breathing difficulty, anxiety, depressed mood, and sleep disturbance (181). A significant portion of approximately 14%, reported an even higher symptom burden, with three or more coexisting symptoms. Two of the most prevalent symptoms reported by this population, regardless of gender, were pain and fatigue (182). Somatic symptoms, referring to physical manifestations of discomfort, pain, or other physically distressing conditions that hinder a patient's functional capacity, are important outcomes that patients wish to address (175). Any symptom that triggers a loss of functional ability is associated with poor functional health and remains a top priority from the patient's perspective (177). Unmanaged, persistent symptoms can significantly compromise the HRQoL of patients (183). The impact of persistent pain has been shown to be profound as it poses the most significant obstacle to performing ADLs and IADLs (182). Pain management is often a recurring theme in patients' discussions of physical health, and thus, it stands as a high-priority health outcome (184).

Chronic conditions such as SIHD and stable angina are examples of high-burden symptoms that significantly impact all aspects of a patient's QoL (185). In a comparative study of more than ten diseases, symptoms associated with CAD were found to exert the second highest impact on functional disability (186). A recent analysis showed that patients with typical angina had poor scores in the physical health component of SF score, as well as patients had much higher anxiety than those without typical angina (187). Taken together, symptom relief should routinely be assessed and managed appropriately in SIHD patients and is a critical outcome that needs to be incorporated in future research that targets SIHD in the older adult population.

*Key Takeaways*

1. *The symptom burden in older adults is multifaceted due to the concomitant presence of multiple chronic conditions. Older adults experience multiple symptoms, which may or may not overlap with SIHD symptoms, such as pain, fatigue, dyspnea, and mood disturbances*.
2. *Symptoms that interfere with the functional ability of older patients are emphasized as a high priority when discussing preferred health outcomes*.
3. *SIHD is a disease that results in high-burden of symptoms that significantly impacts all aspects of older patients’ QoL. Symptoms associated with SIHD considerably impact functional disability, making their management critical in care for older adults*.

#### 5.2.5. Mental health

Mental health serves as an important component in the context of successful aging (171). This is understood not merely as the absence of depressed or negative feelings but also incorporates the presence of positive mental outlooks and robust coping mechanisms (173, 175). These findings are corroborated throughout the literature. For instance, a study assessing the correlation between exercise tolerance and age discovered a robust independent association between high depression scores and age-associated exercise intolerance (180). A comprehensive review by *Pressman et al*. found that patients with a more pronounced negative affect reported their physical symptoms as disproportionately severe relative to their actual disease (188). Conversely, a positive mental outlook was linked with improved health outcomes (189), higher subjective QoL (188), and successful aging (173).

It was proposed that any acute illness might trigger a stress response in older patients, leading to a spectrum of adjustment disorders (190). This captures the maladaptive psychological responses prompted by changes in life circumstances, with diseases playing a significant role. The concept is unequivocally illustrated in the AHRQ evidence report, which found a strong association between developing MI and increased depressive symptoms (191). Typical angina has been shown to elicit higher anxiety in older adults when compared to their counterparts without these symptoms (187). Importantly, older individuals often demonstrate a robust positive reaction to mental adaptability, which encompasses accepting their life circumstances while maintaining a positive outlook on life (176). Such resilience underscores the pivotal role mental health plays in their overall well-being, particularly in the context of managing chronic disease. Consequently, mental health emerges as a significant PRO that should be prioritized in the management of SIHD in the older adult population to ensure adequate QoL. When adverse events occur, interventions to help cope with the stressor should be provided to older patients to ensure adequate recovery of functional abilities.

*Key Takeaways*

1. *Mental health is a critical component of successful aging, encompassing not only the absence of negative feelings but also the presence of positive mental outlooks and robust coping mechanisms*.
2. *Patients with more pronounced negative affect report their physical symptoms as disproportionately severe relative to their actual disease, impacting the perception of health status*.
3. *SIHD has been independently associated with increased depressive and anxiety symptoms as well as acute stress response, which can cause maladaptive psychological responses*.

#### 5.2.6. Cognitive function

There is a known trend of cognitive functional decline with pathologic aging, impacting all facets of cognitive functioning (192). Cognitive functioning serves as a critical pillar of achieving functional independence among older patients and hence maintaining their sense of well-being as well as perceptions of successful aging. Adequate cognitive functioning includes the preservation of memory, the ability to engage in cognitive activities within their community, and the capacity to acquire new skills or experiences (173). In the older population, cognitive dysfunction can span a spectrum that ranges from mild cognitive impairment to more severe forms such as dementia, including Alzheimer's disease (193). Even mild cognitive impairment can notably undermine the ability of older adults to maintain their independence (192, 194). Moreover, the implications of cognitive impairment extend to HRQoL, with both subjective and mild cognitive impairment correlating with poor HRQoL outcomes (195, 196).

An important concept in gerontology is cognitive frailty, which is defined by the International Academy on Nutrition and Aging (I.A.N.A) and the International Association of Gerontology and Geriatrics (I.A.G.G) consensus group as the simultaneous presence of physical frailty and mild cognitive impairment in the absence of dementia or other pre-existing brain disorders. This state of cognitive frailty contributes to increased disease burden and is associated with poorer outcomes (46, 197). When we consider the impact of SIHD or ACS on cognitive function, several studies show that these conditions can indeed have a detrimental impact. For instance, individuals with SIHD often demonstrate poorer cognitive function compared to their counterparts, affecting domains such as memory, attention, and executive function. The exact mechanism of this interaction is multifaceted, with potential contributions from cerebrovascular disease, shared risk factors (198), and the effects of chronic systemic inflammation. Moreover, post-ACS patients may experience a decline in cognitive function, which can influence their functional independence and HRQoL. This highlights the importance of routine cognitive assessment and appropriate management in the context of SIHD, which could subsequently lead to improved outcomes and QoL in these patients.

*Key Takeaways*

1. *Cognitive abilities, including memory, the ability to engage in cognitive activities, and the capacity to acquire new skills, are frequently emphasized in older individuals' perceptions of successful aging*.
2. *Studies have shown that both SIHD and ACS can have a detrimental impact on cognitive function, which in turn can cause functional impedance. Hence, routine cognitive assessment and appropriate management in this patient cohort is a necessity*.

#### 5.2.7. Social support

Social support and meaningful interpersonal relationships are fundamental for older patients to maintain a QoL that is personally fulfilling. This encompasses not only the avoidance of loneliness but also the establishment and continued cultivation of positive connections. Receiving emotional and psychological support from family, friends, colleagues, and others in their social circles, being contributing members of society, and feeling a sense of belonging is essential to their well-being (175, 176, 179, 199). These relationships provide instrumental and emotional support, fostering resilience and adaptability in this population (200). Evidence shows that the social dynamics of the older individual's life can directly impact their HRQoL (183, 201, 202), and subjective social aspects are associated with both subjective well-being as well as positive health affect (173, 203). These findings make it clear that subjective health parameters are interconnected and can mutually influence the overall well-being of the older patient. These complex interactions between social relationships, subjective well-being, and health-related outcomes suggest a need for an integrative and comprehensive approach to the management of CAD in older patients.

*Key Takeaways*

1. *Social support, strong interpersonal relationships, and maintaining roles within the community are important for ensuring adaptability in older adults*.
2. *Social dynamics of an older individual's life can directly impact both the mental health component as well as HRQoL and hence should be taken into perspective when deciding any management strategies*.

### 5.3. Gaps in knowledge

Understanding and acknowledging the need for a patient-centered approach in managing SIHD in the older adult population, several gaps in the current body of research need to be addressed:

1. The categorization of “older adults” at a threshold age of 65 years, might not be an accurate representation of today's older adult populations due to improving health care and increased life expectancy. Studies examining the “old-old” population, those above 85 years and older, and incorporating biologic or physiologic aging are required to understand cardiovascular aging.
2. Despite the acknowledgment of the significance of PROs in geriatric cardiology, they are still under-utilized in research and practice. Systematic reviews or meta-analyses investigating patient-preferred outcomes such as functional independence, cognitive abilities, and mental health in SIHD management, are limited and not externally validated.
3. Clinical trials aimed at enrolling older patients with SIHD must incorporate patient-reported outcomes in their methodology.
4. The development and validation of novel outcome measures that capture older adults' priorities are needed. Current outcome measures may not fully capture the range of patients' experiences and concerns, particularly in the realm of mental health and social functioning.

## 6. Conclusion

The National Academy of Medicine, the European Society of Cardiology, the American College of Cardiology, the American Heart Association, and the American Geriatric Society, strongly advocate for patient-centered care and propose personalized strategies for managing older patients living with SIHD. To optimize cardiovascular care for older patients with SIHD, research evaluating therapeutic outcomes must consider patient preferences and their perceptions of successful aging. These factors should be evaluated within each patient's unique cultural, social, and physical contexts, and weighed against the risks and benefits concerning mortality and morbidity (Figure 2). Geriatric syndromes should be recognized for their significant prognostic implications, and therapeutic interventions should be combined with both preventative and long-term care plans to mitigate these. Discussing therapeutic interventions for SIHD necessitates a comprehensive dialogue about the burden of treatment on the patient, balancing short- and long-term goals identified by the individuals themselves. To ensure a comprehensive and accurate assessment, standardized definitions for patient-reported outcomes in the older population should be the next frontier in clinical research. Taken together, guidelines for chronic coronary disease should not solely focus on managing hard clinical outcomes of SIHD, but rather reflect a more comprehensive person-centered care by incorporating PROs in the approach to management.
